# In vitro inhibition of mumps virus by retinoids

**DOI:** 10.1186/1743-422X-10-337

**Published:** 2013-11-14

**Authors:** Kaitlin J Soye, Claire Trottier, Thomas Z Di Lenardo, Katherine H Restori, Lee Reichman, Wilson H Miller, Brian J Ward

**Affiliations:** 1 Research Institute of the McGill University Health Center, Department of Infectious Diseases, McGill University, Montreal, Quebec, Canada; 2 Segal Cancer Centre, Lady Davis Institute for Medical Research, SMBD Jewish General Hospital, McGill University, Montreal, Quebec, Canada; 3 Montreal General Hospital, 1650 Cedar Ave., Room L10-309, Montreal, Quebec H3G 1A4, Canada

## Abstract

**Background:**

Mumps virus (MuV) is a highly infectious paramyxovirus closely related to measles virus (MeV). Despite the availability of a mumps vaccine, outbreaks continue to occur and no treatment options are available. Vitamin A and other naturally occurring retinoids inhibit the replication of MeV *in vitro*.

**Methods:**

Anti-viral effects of retinoids were observed in cell culture using the myelomonocytic U937, NB4/R4, and Huh7/7.5 cells. Observations of anti-viral effect were quantified using TCID50 analysis. Molecular properties of the antiviral effect were analysed using quantitative RT-PCR and western blot.

**Results:**

The current work demonstrates that retinoids inhibit MuV *in vitro* due to up-regulation of type I interferon (IFN) and IFN stimulated genes. This effect is mediated by nuclear retinoid receptor signalling and RIG-I is required. The antiviral retinoid-induced state makes cells less permissive to viral replication from subsequent challenge with either MuV or MeV for less than 12 hours.

**Conclusions:**

These results demonstrate that retinoids inhibit MuV replication in uninfected bystander cells through a retinoid inducible gene I (RIG-I), retinoic acid receptor (RAR) and IFN dependent manner making them refractory to subsequent rounds of viral replication. These observations raise the possibility that pharmacological doses of retinoids might have clinical benefit in MuV infection.

## Text

The *Paramyxoviridae* are single stranded, enveloped, negative sense RNA viruses. They are among the most important viral pathogens of humans and animals. Many of the *Paramyxoviridae* replicate only in the respiratory epithelium, but *Morbillivirus* and *Rubulavirus* members typically have wider tissue tropism and can cause severe, systemic disease [1]. *Paramyxovirdae* epidemics in virgin populations can be devastating [1]. Vaccines are available for only a small number of the *Paramyxoviridae* and antiviral drugs are not yet available for most of these agents.

Mumps virus (MuV) is a *Rubulavirus* in the *Paramyxoviridae* family. It is the causative agent of mumps [2]. MuV is a highly contagious infection of humans and was historically one of the most common childhood illnesses. The virus infects and replicates in the nasal mucosa and upper-respiratory tract [2]. A transient cell-associated viremia (of mononuclear cells) contributes to systemic viral spread [2]. In young children, MuV infection is typically a mild disease characterized by fever, headache and swelling of the salivary glands. Complications such as meningitis, encephalitis or orchitis may occur. Mumps is a leading cause of acquired sensorineural deafness among children. Rates of post-infectious meningoencephalitis can be 1-10% of clinical mumps cases. Although the fatality rate of mumps encephalitis is low (0.1-0.5% of clinical mumps cases), the risk of permanent neurologic sequelae in encephalitis cases is 25% [3]. Furthermore, MuV infection during the first trimester of pregnancy is associated with a 25% incidence of spontaneous abortion [3].

There is no current treatment for mumps other than supportive care [2]. Vaccination programs in developed countries have markedly increased the average age at which clinical mumps occurs and dramatically reduced the incidence of mumps infection [2]. Unfortunately, large outbreaks have recently occurred in Europe, North America, Australia and Israel [4]–[12].

In the last 2 decades, many studies have documented the beneficial effects of vitamin A supplements on general mortality and/or morbidity in young children in a wide range of developing countries. In 2000, a meta-analysis of eight studies demonstrated an overall 30% reduction in infant mortality attributable to vitamin A supplements [13]–[15]. A surprising spin-off from these vitamin A supplementation studies was the re-discovery that vitamin A ‘treatment’ can significantly decrease the morbidity and mortality associated with acute MeV infection [16]–[19]. The mechanism underlying the positive effects of vitamin A supplements and treatment in measles are not well understood [13]. Since the mid-1990s, the WHO and UNICEF have recommended vitamin A treatment for acute measles in regions of the developing world with high mortality rates [20].

Vitamin A (retinol) is a fat-soluble vitamin. Its natural and synthetic derivatives as well as metabolites are collectively referred to as retinoids [21,22]. Retinol is obtained from the diet as either retinyl esters or carotenoids. Retinoids are required for a wide-range of crucial biological processes including regulation of embryonic development, maintenance of the integrity of epithelial cell surfaces, vision and immunity [23]. The metabolite, all-*trans* retinoic acid (ATRA) is responsible for mediating many of the important biological functions of retinoids [22]. ATRA is the natural ligand for retinoic acid receptors (RAR), which form heterodimers with the retinoid X receptors (RXR) within the nucleus [24]. RAR-RXR heterodimers bind to retinoic acid response elements (RARE) on the promoters of target genes to activate transcription when bound by ligand [21,22,24]. The protein products of retinoid-responsive genes are responsible for exerting the effects of retinoids in the cell.

Retinoids have been shown to play a role in innate immune responses and to regulate the expression of a number of interferon stimulated genes [25]–[27]. Of particular interest among the retinoid-responsive genes is the type I interferon (IFN) pathway. A powerful trigger for type I IFN production is the recognition of virus-associated molecular patterns by pattern recognition receptors [28]. These cytokines trigger a rapid and strong innate defense against many viruses, leading to the transcription of several hundred ISGs controlled by the IFN-stimulated gene factor 3 (ISGF3) complex [29].

Of particular importance to the current work, retinoids have specifically been implicated in regulating expression of the ISG (Interferon Stimulated Gene) retinoid-inducible gene I (RIG-I) and IFN regulatory factor 1 (IRF-1) [30]–[39]. RIG-I is a pattern recognition receptor that was originally understood to detect 5’-triphosphorylated, single-stranded RNA [40]–[42] and is expressed at a basal level in many cell types. The current consensus is that the minimal requirement for RIG-I activation is a blunt-ended base paired RNA 10-20 bp long with a 5’ triphosphate [43]. It can initiate the production of type I IFN and is itself an ISG [44]. IFN has been reported to induce RIG-I expression by causing the IRF-1 transcription factor to bind to the RIG-I promoter [45].

Anti-MeV effects of retinoids have been observed in a number of primary human cells and cell lines of diverse tissue origin [46]–[48], including the myelomonocytic U937 cells, which were an important model for our work with MuV presented herein. We hypothesize that ATRA treatment during other viral infections would also have an antiviral effect. We set out to test whether or not MuV replication could be inhibited by retinoids. Based on our previous studies, we hypothesize that retinoids would inhibit MuV replication *in vitro* and that this inhibition would depend upon RAR signalling, type I interferon and functional RIG-I.

## Results

### Mumps virus can be inhibited in vitro

**Figure 1:**
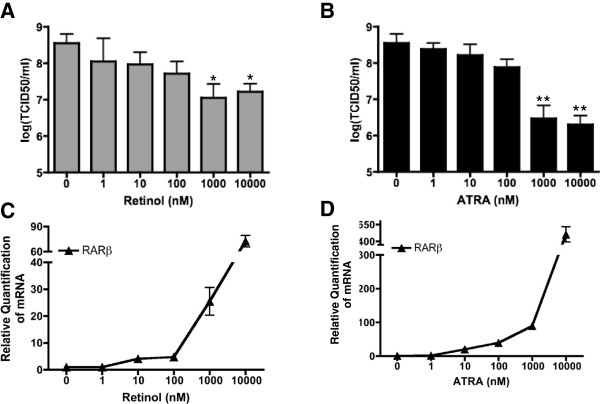
***In vitro *inhibition of mumps virus by retinoids. (A) (B)** U937 cells were infected with MuV at an MOI of 0.01 and treated with increasing doses of retinol or all-*trans* retinoic acid (ATRA) as indicated. Whole cell lysates were harvested after 48 hours and viral titers were measured by TCID_50_. **(C) (D)** RNA was extracted from parallel U937 cultures treated with increasing doses of retinol or ATRA and analyzed for RAR-β expression by qPCR. Data presented reflect three experiments performed in triplicate (N = 3). *p < 0.05, **p < 0.01.

U937 cells are neoplastic and histiocytic progenitors of monocytes that have been extensively used in immunological studies [49] including investigation of interferon pathways during MuV infection [50]–[52]. In these cells, increasing doses of retinol resulted in a significant inhibition of MuV replication as quantified by TCID_50_ (Figure 1A). Significant inhibition was achieved at concentrations as low as 1 μM, a dose at which increased expression of the retinoid responsive gene RARβ is readily observed (Figure 1C) [53]. Treatment of U937 cells with increasing doses of ATRA was even more effective as an inhibitor of MuV output (Figure 1B) and in the induction of RARβ mRNA expression (Figure 1D) [53]. All subsequent investigations of the antiviral effect of retinoids on MuV were performed using ATRA at a dose of 1 μM.

### Retinoid treatment enhances IFN signalling

**Figure 2:**
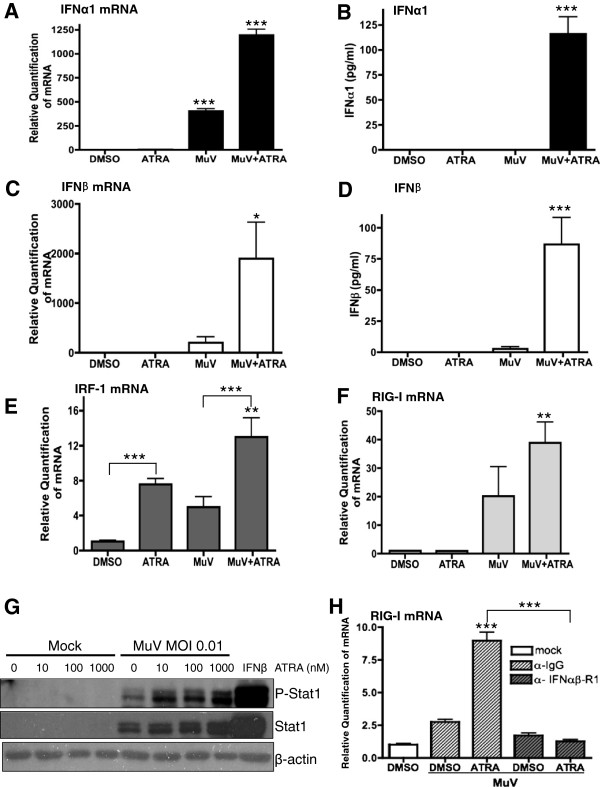
**Type I interferon signaling is required for the induction of the retinoid anti-MuV response.** U937 cells were infected with MuV at an MOI of 0.01 and treated with 1 μM ATRA or DMSO. 48 hr post-infection, RNA was extracted and analyzed for IFNα1 **(A)**, IFNβ (C), IRF-1 **(E)** and RIG-I **(F)** expression by qPCR. Supernatants were analyzed by ELISA for IFNα1 **(B)** or IFNβ **(D)** protein. **(G)** U937 cells were treated with increasing doses of ATRA (0-1000nM) and either mock infected or infected with MuV at an MOI 0.01. Protein was isolated from whole cell extracts and analyzed by western blot for phospho-STAT1 (Y701), total STAT1 or β-actin. **(H)** U937 cells were infected with MuV at an MOI of 0.01 and treated with 1 μM ATRA or DMSO and isotype control antibody or IFNAR2 antibody. 24 hr post-infection RNA was extracted and analyzed for RIG-I expression by qPCR. Data presented reflect three experiments performed in triplicate (N = 3). *p < 0.05, **p < 0.01, ***p < 0.001.

The innate immune response is thought to be responsible for the initial control of infectious agents. It has long been known that up-regulation of the type I IFN response functions in an auto-response feedback loop that is critically important for antiviral responses. In the U937 model, MuV infection alone is able to induce the expression of IFNα1 mRNA (Figure 2A). However, ATRA treatment of MuV infected cells synergistically increases the expression of IFNα1 mRNA and supernatant protein levels (Figure 2A-B). IFNβ mRNA expression and protein levels are also synergistically increased by the combined treatment of ATRA and MuV infection (Figure 2C-D).

The increased type I IFN production leads to the expression of ISGs. In the U937 model, IRF-1 mRNA expression is significantly increased over control by ATRA treatment alone (Figure 2E), in agreement with our previous work [47] and the literature [30,38,39]. However, treatment of MuV infected cells with ATRA further increases the expression of IRF-1 mRNA (Figure 2E). This combined treatment (MuV + ATRA) resulted in a robust increase in RIG-I mRNA expression (Figure 2F). The mRNA levels of two other IFN-responsive genes, IRF-7 and MDA-5, also showed similar patterns of increased expression in response to MuV + ATRA (data not shown). In addition to the regulation of ISG expression, treatment of MuV infected U937 cells with ATRA also increased STAT1 activation as indicated by phosphorylation of tyrosine 701 (Figure 2G).

The increased expression of these ISGs can be attributed to the increased activation of the type I IFN pathway. When a monoclonal antibody specific to IFNα/β receptor 1 was used to prevent IFN signalling during MuV + ATRA treatment, ISG mRNA expression was blocked, as demonstrated by RIG-I mRNA (Figure 2H). This observation demonstrates that IFN signalling is required for the retinoid-MuV antiviral response.

### Functional nuclear retinoid receptors mediate antiviral activity of retinoids

**Figure 3:**
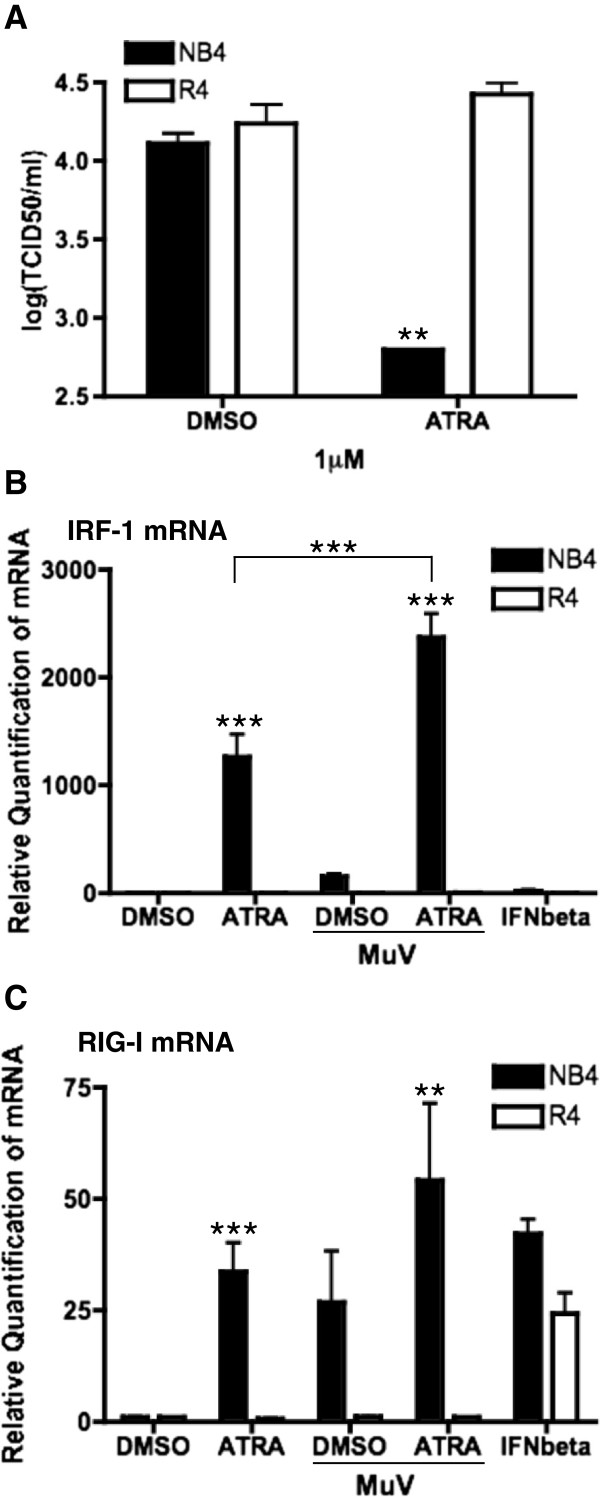
**Retinoid signaling is required for the inhibition of MuV *in vitro*.** NB4 and R4 cells were infected with MuV at an MOI of 0.01, treated with 1 μM ATRA or DMSO and/or treated with 1000 U/mL IFNβ (PBL Interferon Source, New Jersey) as indicated. **(A)** Whole cell lysates were harvested after 48 hours and viral titers were measured by TCID_50_. RNA was extracted and analyzed for IRF-1 **(B)** and RIG-I **(C)** expression by qPCR. Data presented reflect three experiments performed in triplicate (N = 3). *p < 0.05, **p < 0.01, ***p < 0.001.

To determine whether the antiviral activity of retinoids requires nuclear receptor signalling, we utilized the well-characterized NB4/R4 cell model (retinoid responsive versus retinoid unresponsive) [54]. NB4 cells respond to ATRA at pharmacologic concentrations, while the NB4 subclone R4 is completely resistant, regardless of the concentration [54,55]. Both NB4 and R4 cells were readily infected with MuV. In NB4 cells, 1 μM of ATRA was able to inhibit MuV output but had no effect in R4 cells (Figure 3A). At this concentration, the level of inhibition observed was unlikely due to retinoid-driven differentiation of the NB4 cells [46,48]. Like the U937 cells, expression of the ISG, IRF-1, was also increased in NB4 cells exposed to ATRA alone but was higher in cells exposed to MuV + ATRA infection (Figure 3B). IRF-1 mRNA expression was very low during MuV infection alone in this model. In the retinoid-unresponsive R4 cells, IRF-1 expression was not seen either with ATRA treatment alone or in response to MuV + ATRA (Figure 3B). Exogenous IFNβ treatment alone was not able to induce the expression of IRF-1 in either cell line, suggesting the requirement of ATRA for IRF-1 expression.

RIG-I mRNA expression was also significantly increased by the combined treatment of MuV + ATRA in NB4 cells (Figure 3C). Both MuV alone and ATRA alone increased the expression of RIG-I over mock treatment, but the expression was greatly enhanced by combined treatment. Neither ATRA, nor MuV + ATRA induced the expression of RIG-I mRNA in R4 cells (Figure 3C). When treated with exogenous IFNβ, both NB4 and R4 cells increased the expression of RIG-I mRNA suggesting that IFN signalling is functional in both cell lines (Figure 3C). Expression of other ISGs, including IRF-7 and MDA-5, showed a similar pattern of up-regulation in NB4 cells and no response in R4 cells (data not shown).

As a further confirmation of the role of RARα mediated signalling in the retinoid-MuV antiviral response, treatment of U937 cells with RO 41–5253, a specific RARα antagonist, reversed the impact of ATRA on MuV replication and reduced the expression of the ISGs in response to MuV + ATRA (data not shown).

### RIG-I is required for the retinoid-induced antiviral response

RIG-I is both retinoid responsive and IFN stimulated. It was clearly up regulated in our *in vitro* model systems in response to MuV + ATRA (Figures 2F, 3C). To investigate the requirement of RIG-I signalling in the cellular response to combined MuV + ATRA exposure, we used the Huh7 cell line, which is derived from a human hepatocellular carcinoma and has been used extensively in hepatitis C virus (HCV) research [56,57]. Of particular interest for our studies, an Huh7 subclone (Huh7.5) has a point mutation in the first CARD domain of RIG-I, rendering the protein non-functional [57,58].

**Figure 4:**
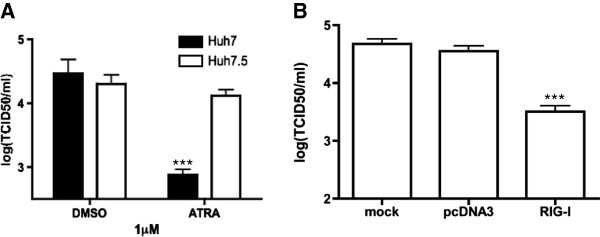
**RIG-I is required for the inhibition of MuV by retinoids. (A)** Huh7 and Huh7.5 cells were infected with MuV at an MOI of 0.01 and treated with 1 μM ATRA or DMSO. Whole cell lysates were harvested after 48 hours and viral titers were measured by TCID_50_. **(B)** Huh7.5 cells were transfected with mock, pcDNA3.1 or pRIG-I-myc and incubated overnight. Following transfection, the cells were infected with MuV at an MOI of 0.01 and treated with 1 μM ATRA. Whole cell lysates were harvested after 48 hours and viral titers were measured by TCID_50_. Western blotting was not performed since Huh7.5 cells produce a defective RIG-I protein that cannot be distinguished from the wild-type protein by commercially-available antibodies. Data presented reflect two experiments performed in triplicate (N = 2). ***p < 0.001.

We turned to the Huh7/7.5 model to demonstrate the importance of RIG-I rather than using RNA interference (RNAi) after initial experiments demonstrated that both control and RIG-I specific siRNA were sufficient to induce the expression of RIG-I and other interferon stimulated genes (data not shown, also demonstrated in [59,60]). In MuV infected Huh7 cells treated with ATRA, virus output was significantly reduced (Figure 4A) but ATRA had no effect on MuV replication in the Huh7.5, RIG-I non-functional cells (Figure 4A).

It has recently been demonstrated that RIG-I complementation in Huh7.5 cells can restore the IRF3 pathway, making these cells less permissive to Sendai virus (SeV) infection [58]. This observation suggests that the non-functional RIG-I encoded in the Huh7.5 cells can be complemented by exogenous expression of the protein. When RIG-I was transfected into the Huh7.5 cells, inhibition of MuV replication was restored (Figure 4B). These data demonstrate the requirement of RIG-I in the retinoid-MuV antiviral response.

### Antiviral response is created in uninfected bystander cells

To determine whether or not a bystander effect was induced following MuV infection, we repeated key experiments using 0.02 μm-pore membrane transwell tissue culture inserts (depicted in [48] and [47]). In these experiments, the inner-chamber U937 cells could be exposed to the products of infection in the outer-chamber cells without direct contact with either MuV itself or the MuV-infected cells. We confirmed that MuV was not able to cross the membrane by TCID_50_ assay of the inner-chamber cells in each experiment.

**Figure 5:**
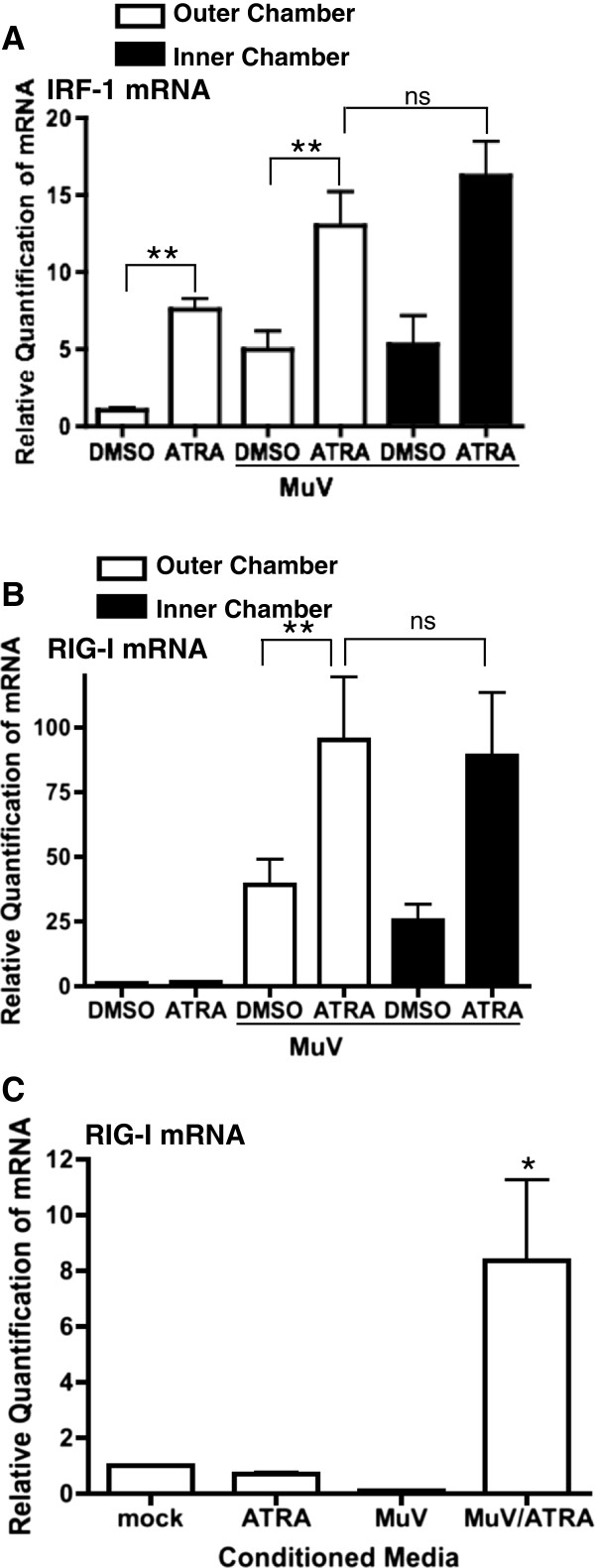
**Retinoids induce an anti-MuV state in the uninfected, bystander cells.** U937 cells were infected with MuV at an MOI of 0.01 in the presence of 1 μM ATRA or DMSO. Transwell membrane inserts with 0.02 μm pores were used to separate the infected cells in the outer chamber from the uninfected, bystander cells in the inner chamber [48]. Cells from control wells (no membrane insert), outer and inner chamber bystander cells were harvested after 48 hours and IRF-1 **(A)** and RIG-I **(B)** mRNA were measured by qPCR. As indicated on the Figure, outer chamber cells infected by MuV are represented by open bars and inner chamber (uninfected) cells are represented by the filled bars. **(C)** Conditioned media from the control and transwell inner chambers were applied to fresh U937 cells. After 24 hours of incubation with the conditioned media, RNA was extracted and RIG-I expression was analyzed. Data presented reflect three experiments performed in triplicate (N = 3). * p <.02,**p < 0.01.

ATRA-stimulated ISG expression was just as strong in the inner-chamber (uninfected) as the outer-chamber (infected) cells despite the absence of active infection. Specifically, we found strong up-regulation of mRNA expression for IRF-1 (Figure 5A) and RIG-I (Figure 5B), as well as MDA-5 and IRF-7 (data not shown), in the inner-chamber cells.

When the supernatant (or conditioned media) from the inner-chamber bystander U937 cells was applied to fresh cells, we observed a striking induction in the expression of these same ISGs as shown for RIG-I (Figure 5C).

### Bystander cells are protected from infection

**Figure 6:**
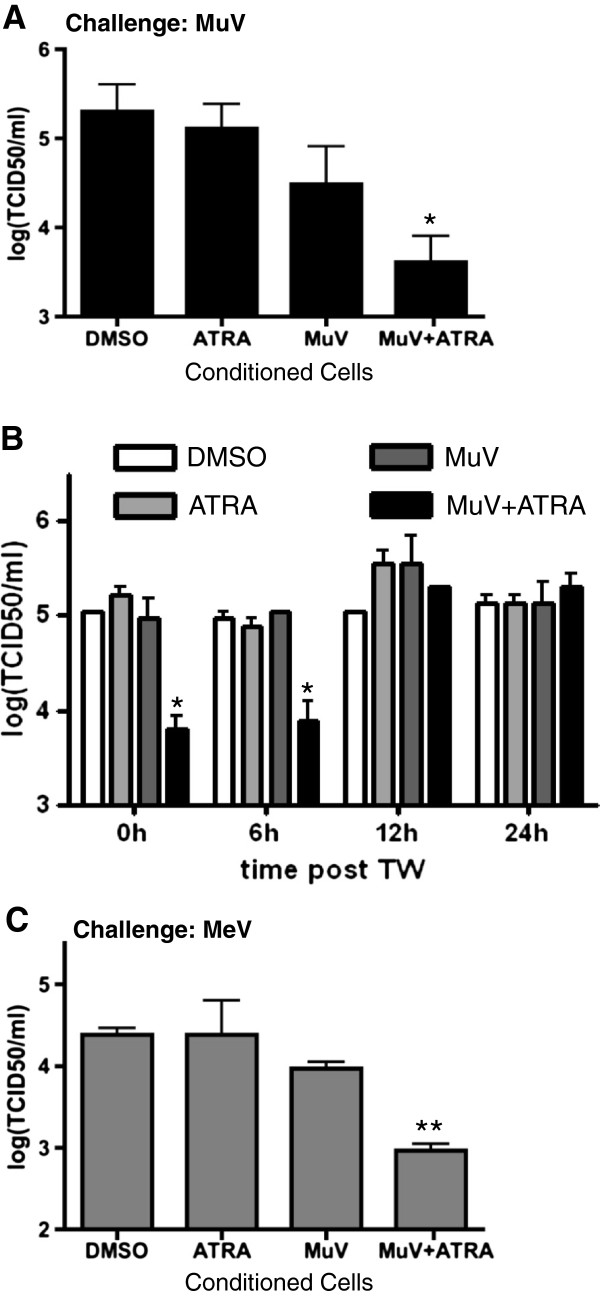
**Antiviral state is short-lived and virus non-specific. (A)** The inner chamber bystander cells were harvested and challenged with MuV at an MOI of 0.1. Whole cell lysates were harvested after 48 hours and viral titers were measured by TCID_50_. **(B)** To assess the duration of the anti-viral state, U937 cells were suspended in TW inner-chamber conditioned media and incubated for the indicated time at 37°C then challenged with MuV at an MOI of 0.1. Whole cell lysates were harvested after 48 hours and viral titers were measured by TCID_50_. **(C)** The inner chamber bystander cells were harvested and challenged with MuV at an MOI of 0.1. Whole cell lysates were harvested after 48 hours and viral titers were measured by TCID_50_. Data presented are representative of two-three experiments performed in triplicate (N = 2-3). *p < 0.05, **p < 0.01.

To determine whether or not the uninfected inner-chamber, bystander cells would have reduced susceptibility to future infection these cells were harvested and challenged with MuV at an MOI of 0.1 immediately following incubation in the transwell. Compared with control cells not treated with ATRA and exposed to the products of MuV infection, the inner-chamber cells were relatively resistant to MuV replication (one log reduction in MuV titres produced, Figure 6A). This relatively refractory state persisted for up to 6 hours but was lost at 12 hours (Figure 6B). These data suggest that the antiviral state created in the bystander U937 cells is short lived.

When inner-chamber bystander cells treated with ATRA and exposed to the products of MuV infection were challenged with MeV at an MOI 0.1 MeV replication was also reduced by at least 1 log compared to untreated controls or cells treated with only ATRA or exposed to the products of outer-chamber MuV infection (Figure 6C). The antiviral state induced in these cells was not virus-specific.

## Discussion

The potential role of individual micronutrients in specific infectious diseases has been the subject of considerable interest for decades (reviewed in [61]). To our knowledge, retinol (Vitamin A) is currently the only micronutrient routinely used to ‘treat’ a viral disease. In fact, both vitamin A supplementation and therapy appear to have significant clinical benefit in natural MeV infection [16]–[19,21]. However, the effects of vitamin A on viral infections have been highly variable and at times, completely contradictory.

Although reduced mortality from diarrheal disease is associated with vitamin A supplements in children of the developing world this benefit appears to be due largely to milder bacterial infections [14,62,63]. In Mexican children receiving vitamin A supplements, the incidence of Norovirus diarrhea was reduced but gut viral titres and the period of virus shedding in these children were both significantly increased [64].

In human immunodeficiency virus (HIV) infection, pre-antiretroviral treatment (ART) studies suggested that low serum retinol levels were associated with rapid progression of acquired immunodeficiency syndrome (AIDS) but later studies showed little-to-no impact of supplements on disease progression or survival (reviewed in [65]). Perinatal vitamin A supplements in HIV-positive women can improve the survival of the seronegative children but can increase mother-to-child HIV transmission [65], possibly through increased viral loads in breast milk [66]. *In vitro*, retinoids have been found to both increase and decrease HIV replication in different model systems [67,68].

Patients infected with Hepatitis C virus (HCV) and treated with 9-*cis* retinoic acid or ATRA in combination with pegylated IFNα have lower viral loads [69,70]. In contrast, supplements do not increase viral clearance in human papilloma virus (HPV)-infected women [71].

Both vitamin A supplementation and treatment have either no or negative effects on respiratory tract infections including the common paramyxovirus, respiratory syncytial virus (RSV) [72]–[74]. Studies with another paramyxovirus have shown that vitamin A deficient chickens suffer increased morbidity from Newcastle disease virus (NDV) [75]–[77]. Using the paramyxovirus most closely related to measles, our group has demonstrated that canine distemper virus (CDV)-infected ferrets treated with vitamin A develop less severe disease [78]. In aggregate, these observations suggest that vitamin A and its derivatives may play an important role in antiviral responses but demonstrate clearly that mechanistic studies are essential to fully understand and exploit this potential.

**Figure 7:**
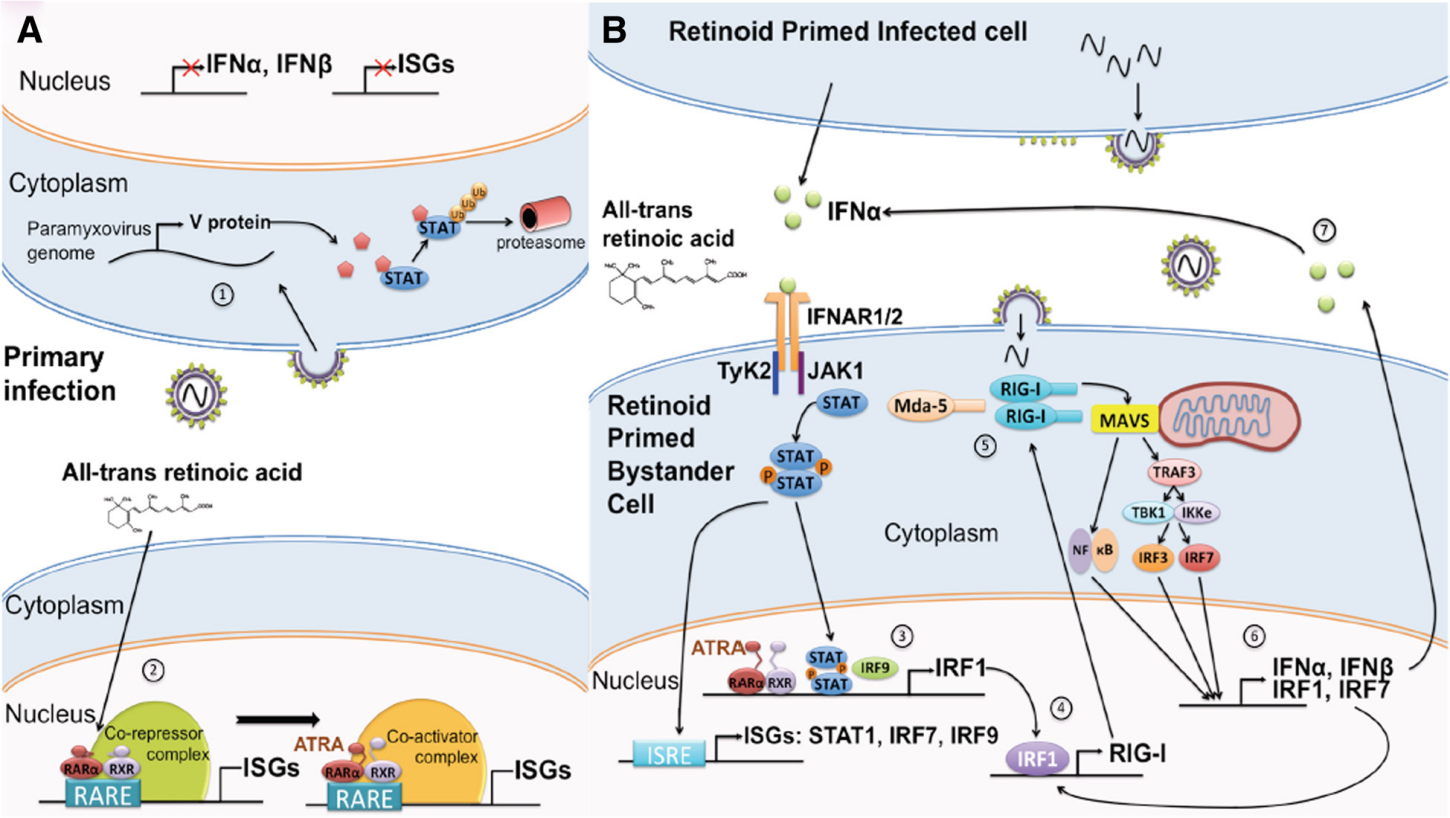
**Retinoid action during paramyxovirus infection. (A)** 1) Jak/STAT signaling is inhibited as a result of viral infection and ATRA treatment is unable to effectively promote unregulation of ISGs and type I IFN production. 2) Cells exposed to the products of the primary infection and primed with ATRA can more efficiently secrete type I IFN upon infection. **(B)** 3) Exposure to ATRA and type I IFN in the supernatant signals through the Jak/STAT pathway with the combination of nuclear localization of STAT1/2 heterodimer and activation of the RAR/RXR complex results in the transcription of multiple ISGs, importantly IRF1. For simplicity, only IFNα and its receptor IFNAR1/2 are shown to promote Jak/STAT signaling. 4) RIG-I expression is greatly up-regulated under these conditions, by IRF1 binding to the interferon response element (IRE) on its promoter. 5) Increased cytoplasmic quantities of RIG-I provide protection in the bystander cell upon virus infection. RIG-I detects viral RNA and associates with the adaptor protein MAVS, which initiates downstream activation of NFκB and IRF3/7. Nuclear translocation to their respective promoter elements induces transcription of antiviral genes, i.e., type I IFN and IRF1/7 production. It is not clear what role the second cytoplasmic RNA helicase (mda5) plays in this cascade although mda5 has been implicated in responses to several Paramyxoviridae. 6) These proteins feedback onto their respective pathways and promote RIG-I expression, further improving the antiviral state of the cell. 7) Increased production of type I IFN from the retinoid-primed bystander cell provides both an autocrine and paracrine signal to further protect the uninfected cells in culture.

Previously we have shown that retinoids can inhibit MeV replication *in vitro* via retinoid nuclear receptor activating type I IFN signalling [46,48]. We hypothesized that ATRA treatment during MuV infection may also inhibit MuV replication *in vitro*. We further sought to determine if the retinoid-MuV antiviral response would require type IFN signalling, RAR signalling and functional RIG-I. The current work demonstrates that ATRA similarly exerts anti-viral effects on MuV. We believe that these effects are not virus-specific, but rather extend to multiple members of the Paramyxovirus family or more broadly, to viruses that are detected by RIG-I. Figure 7 depicts our current understanding of retinoid action on Paramyxovirus infection. In Figure 7A, ATRA alone has no protective capacity on initially infected cells. These cells will produce the same amount of virus as untreated cells and ultimately, will die as a result of infection. However, the initially uninfected cells in the culture are primed for ISG expression by ATRA treatment through activation of the nuclear retinoid receptors. In Figure 7B, retinoid-primed cells effectively up-regulate ISG expression and type I IFN production upon viral infection. The combination of type I IFN and ATRA induces RIG-I expression in uninfected bystander cells, further improving the innate anti-viral response. ATRA is essential for initiating positive feedback through RIG-I activation and type I IFN pathways, which protects uninfected cells.

In the current work, we used a variety of *in vitro* models to extend our central observation of retinoid-induced antiviral effects to MuV (Figures 1A, 1C, 3A, 4A). Although the cell lines used in this work varied in their overall sensitivity to retinoids (NB4 > U937 > Huh7>> R4), all supported the growth of MuV. Retinoid-induced suppression of MuV replication could be demonstrated in all but the R4 cells. Retinol (ROH) is the form of vitamin A found in the circulation at concentrations up to 2 μM [79]. The degree of inhibition of MuV replication was much greater using ATRA, a natural derivative of ROH and ligand that binds directly to nuclear receptors. ATRA is generally found in the intracellular space, but can be found in the serum in the 5–10 nM range [79]. As a result, we believe the mechanisms that we have documented *in vitro* to be potentially active *in vivo*. Indeed, the outcome of any infection is essentially a ‘race’ between pathogen replication and the developing immune response. In this context, it is plausible that the modest reduction in the rate of MuV replication that we observed with retinoid ‘treatment’ *in vitro* could translate into clinical benefit during natural disease, as occurs with vitamin A treatment in natural MeV infection. To our knowledge, there has not yet been any attempt to use retinol (or other retinoids) to modulate the course of mumps infection. Unfortunately, there is no animal model for mumps in which this possibility can be directly tested.

The antiviral state created by the combination of MuV infection and ATRA treatment was ultimately generated by the expression of type I interferon. We have demonstrated that the combination of MuV + ATRA leads to transcription of IFN genes and at least additive increases in IFNα1 and IFNβ levels in culture supernatants, as well as enhanced transcription of ISGs (Figure 2A-F). Increasing doses of ATRA in the context of MuV infection led to marked increases in STAT1 activation (Figure 2G) and, when type I IFN signalling was blocked, the antiviral state is lost (Figure 2H). MuV normally escapes type I IFN control by targeting STAT1 for proteasomal degradation. Variations in the V protein sequence can decrease the efficiency of proteosomal targeting of STAT1, [80] resulting in differing sensitivity to type I IFNs and potentially the IFN dependent antiviral state produced by retinoid treatment. We are currently collecting wild-type MuV isolates to correlate retinoid sensitivity with V protein sequence to better understand this apparent paradox. At the current time, we also cannot fully explain differences in ATRA-induced up-regulation of RIG-I expression between the U937 and NB4 cells other that to postulate greater retinoid sensitivity in the RIG-I promoter of the former line. It is also possible that the timing of sample collection contributed to these results. Similarly, the timing of sampling may underlie the up-regulation of RIG-I mRNA in NB4 cells in response to IFNβ stimulation despite the apparent absence of IRF-1 induction (Figure 3B/C). Time course studies are currently underway to address these issues.

We further demonstrate that nuclear retinoid receptor signalling was also central to the antiviral effect of retinoids against MuV. Although it is possible that more than one nuclear receptor may be involved, our current data suggest that RARα plays an important role in mediating the antiviral effects against MuV. In our NB4/R4 model, RAR signalling was not only required for the antiviral effect (Figure 3A), it was essential for the expression of ISGs that contribute to the antiviral response (Figure 3B-C).

Finally, we demonstrate a similar retinoid signalling mechanism in response to MuV + ATRA (Figures 2E, 3C, Figure 4A). Most convincing, we have shown that overexpression of RIG-I in Huh7.5 cells with non-functional RIG-I signalling, can reinstate the retinoid-induced inhibition of MuV. The results in the Huh7.0/7.5 model are particularly interesting because MuV output does not differ greatly at 48 hours, suggesting that intact RIG-I signalling (by itself) does not play a major role in limiting viral replication. However. transfection of a functional RIG-I clearly restores retinoid responsiveness in this model. At least some of this paradox may be explained by the 48-hour time-point used for most experiments. Indeed, MuV output was lower in the Huh7.0 than Huh7.5 cells for the first 24–36 hours (data not shown). The 48 hour time-point was chosen for our experiments because retinoid effects were most obvious at this time. These findings are very similar to our observations with measles virus in the Huh 7.0/7.5 model where transfection of a dominant negative RIG-I eliminates the anti-viral activity of retinoids in the Huh 7.0 cells and transfection of a functional RIG-I gene into Huh 7.5 cells restores activity [47].

The Huh7.0/7.5 data are also intriguing because they suggest a larger role for RIG-I in defending against MuV than would have been predicted from the literature to date. It is widely thought that the double-stranded RNA sensor mda-5 is the primary target of the MuV V protein [81,82] and that RIG-I may respond primarily to Paramyxovirus defective interfering particles [83]. For several Paramyxoviruses, mda-5 signalling is inhibited by direct binding of the V protein and conserved residues in the helicase [82]. More recent data raises the possibility that Paramyxovirus V proteins may also target RIG-I indirectly by binding to laboratory of genetics and physiology 2 (LGP2) [84] Mutations in the carboxy-terminal domain of the V protein can result in a reduction or total loss of this interference [81]. In both NB4 and U937 cells, mda-5 expression is also increased by ATRA alone (data not shown). We are currently collecting wild-type (WT) MuV isolates to assess their susceptibility to retinoid-induced suppression and to correlate this suppression with V protein mutations. Our preliminary data (4 low-passage isolates to date) suggest that sensitivity to retinoid-induced suppression varies widely in WT MuV (50% suppressible) It is also interesting that retinoid sensitivity has been maintained in the two initially sensitive WT isolates despite repeated *in vitro* passage in Vero cells in our laboratory.

The antiviral state created by MuV + ATRA was most profound in the initially uninfected bystander cells (Figure 5A-B) and could be transferred to fresh cells via the conditioned media leading to up-regulation of ISG expression (Figure 5C). Not surprisingly, since type I IFN responses are innate and non-specific, cells exposed to conditioned media from MuV + ATRA cells were relatively resistant to subsequent challenge with either MuV or MeV for less than 12 hours (Figure 6A-C). This last observation is consistent with the immediate and short-lived antiviral effects of type I IFNs [85].

The *Paramyxoviridae* including MeV, MuV, RSV, CDV, phocine distemper virus, Nipah virus and Hendra virus are among the most important human and animal pathogens. Commercial vaccines are not yet available for many of these viruses, and antiviral drugs are typically of little use [86]. Some of these viruses can have extraordinarily high mortality rates (for example, CDV in naïve seals and dogs, Nipah and Hendra viruses in man) [87]. The clinical evidence of benefit from retinoid therapy of MeV infection in children and CDV infection in ferrets is strong [17]–[19,78]. Our *in vitro* data suggest that ATRA may be far more potent that retinol in mediating antiviral effects. Our mechanistic studies in different tissue culture models of MuV infection suggest that common signalling pathways mediate these effects [46]–[48]. However, high doses of vitamin A in children with RSV infection have no benefit and may even cause harm [74,88]. In aggregate, these clinical and laboratory observations support further studies of the efficacy and mechanism of action of retinoids against a wider range of respiratory viruses in more sophisticated animal models, such as primates, or even clinical studies. It would be of particular interest to use retinoids other than retinol, ATRA in particular, in these latter studies to achieve more effective inhibition of viral replication. This conclusion is further supported by a recent study demonstrating that several synthetic retinoid analogues have much greater capacity to interfere with human herpes virus 8 (HHV8) replication *in vitro* than retinol [89].

## Conclusions

In conclusion, this work has demonstrated that MuV can be inhibited *in vitro* by retinoids. This antiviral effect required RAR signalling, type I IFN signalling and functional RIG-I. The antiviral response was created in the initially uninfected bystander cells and was both short-lived and cross-protective against subsequent MuV or MeV challenge. This is the first work to demonstrate the antiviral effect of vitamin A on MuV and may contribute to better treatment options for MuV. We propose that IRF-1 is recruited to the RIG-I promoter under the influence of ATRA alone, and is required for the induction of RIG-I (47). In these models systems therefore, ATRA inhibits MuV replication through the RARα-dependent regulation of RIG-I and IRF-1 and via an IFN feedback loop.

## Methods

### Cells, reagents and viruses

All cell cultures were maintained at 37°C in a 5% CO_2_ humidified incubator. U937 (ATCC, #CRL-1593.2), NB4 (M. Lanotte, INSERM UMR-S 1007, Paris, France) and R4, Huh7 and Huh7.5 (courtesy C. Richardson, Dalhousie University, Halifax, NS), Vero cells (ATCC, #CCL-81) were maintained as described in [47]. Retinol and All-trans retinoic acid (ATRA) (Sigma-Aldrich Fine Chemicals, Oakville, ON) stock solutions of 10^−2^ M were prepared in 100% DMSO and further dilutions were performed using RPMI. DMSO at equivalent final dilutions was used in all experiments as a control. All retinoids were stored in opaque eppendorf tubes at −80°C. The Jones MuV strain (ATCC, #VR-365) is a tissue culture-adapted virus that was, according to the supplier’s web-site, extensively passaged in chicken embryos and Vero cells prior to purchase. Our MuV stock was initially plaque purified and then grown by infecting Vero cells with a maximum passage of three times from the original purchase (ATCC, #CCL-81) at a multiplicity of infection (MOI) of 0.001 at 33°C in a Cell-Stack 10 (Corning, Corning, NY). Harvested virus was concentrated by centrifugation at 15,752 x g for seven hours at 4°C in a fix-angle rotor, the pellet was resuspended in RPMI with gentle pipetting. The Chicago-1 MeV strain is a tissue culture-adapted genotype D3 virus (courtesy of W. Bellini, CDC, Atlanta, GA). MeV stock was grown as described in [47].

### Cell culture infections

Cell lines were infected with MuV at the indicated MOIs. Media was removed and virus diluted in Hanks’ Balanced Salt Solution with calcium and magnesium (Wisent, St-Bruno, QC). The virus was incubated with the cells for 1.5 hours, with gentle rocking at 15-minute intervals. The virus was removed and cells were resuspended in RPMI 1640 supplemented as previously described [46]–[48] using the specific MOIs and time points indicated in the figure legends and incubated at 37°C/5% CO_2_.

### Quantitative RT-PCR

RNA was extracted using Trizol (Invitrogen by Life Technologies, Burlington, ON) as per the manufacturer’s instructions, and treated to remove possible genomic DNA contamination with Turbo DNAse (Ambion, Austin, TX). For experiments in which antibodies were used to block type I IFN signalling, an RNeasy Mini kit was used to extract RNA (Qiagen, Mississauga, ON). Equal quantities of RNA were reverse-transcribed into cDNA for qPCR analysis using random primers. FAM-labelled TaqMan primer-probe assays for the following genes were obtained from ABI (Applied Biosystems by Life Technologies, Carlsbad CA): RIG-I, RARβ and IRF-1. The level of gene expression in untreated cells was used for calibration. Vic-labeled hGAPDH was used as the endogenous control.

### Transwell

Transwell experiments (TW) were performed as previously described [47,48]. Briefly, TW membranes inserts with 0.02 μM pores served to separate infected cells in the outer chamber from the uninfected bystander cells of the inner chamber. Wells with no transwell inserts were used for control cultures. Preliminary experiments demonstrated that the presence/absence of the TW membrane had no impact on measured outcomes under control conditions.

### Conditioned media

Supernatants were collected from TWs and used to treat fresh U937 cells. After 24 hours of incubation with the TW conditioned media, RNA was extracted and RT PCR performed. These samples were analyzed by qPCR for the expression of RIG-I.

### Blocking antibody

Supernatants were collected from TWs and used to treat fresh U937 cells. These fresh cells were treated with anti-IFNAR2 blocking antibody (20 μg/μL, PBL Biomedical Laboratories, Piscataway, NJ) or isotype control antibody for one hour before infection and for the subsequent 24-hour incubation with the conditioned media. These samples were analyzed by qPCR for the expression of RIG-I.

### Western blotting

Cells were infected with MuV and/or treated with ATRA at the indicated doses. 48 hours post infection, protein was harvested as previously described in [47]. The membranes were incubated in 5% non-fat milk or 5% BSA for 1 hour and incubated overnight at 4°C with primary antibody. Primary antibodies used were against phospho-STAT1 (Y701) (1/1000, BD Bioscience), Total STAT1 (1/1000, BD Bioscience) and β-actin (1/10000, Sigma). Following overnight incubation, membranes were washed three times for 10 minutes in TBS/0.1% Tween, incubated with secondary antibody (1/10000, GE Healthcare) at room temperature for 30 minutes, and washed three times for 10 minutes. The peroxidase-conjugated secondary antibodies were developed using a chemiluminescence kit according to the manufacturer’s instructions (GE Healthcare).

### Transfection

Huh 7.5 cells were seeded at 1.5 × 10^5^ cell/mL, then were transfected with 3 μg of the RIG-I construct in a pcDNA3 plasmid (gift from J. Hiscott) or empty vector using a 3:1 ratio of FuGENE 6 (Roche, Toronto, ON) as per the manufacturer’s instructions. At 18 hours post-transfection, cells were infected with MuV MOI 0.01 and at 48 hours post infection the cells and supernatants were quantified using plaque assay as previously described [46].

### Viral challenge of bystander cells

Bystander cells from the TW inner chambers were pooled according to treatment and resuspended in Hanks’ Balanced Salt Solution with calcium and magnesium (Wisent, St-Bruno, QC). Cells are infected with MuV or MeV at MOI 0.1 as described above and previously [46]–[48]. These cells were resuspended in RPMI 1640 (Wisent, St-Bruno, QC) supplemented with 10% heat-inactivated FBS (Wisent, St-Bruno, QC) and 0.1% gentamicin and incubated for the indicated time at 37°C/5% CO_2_.

### Tissue culture infectious dose_50_ (TCID_50_)

MuV concentrations were quantified by TCID_50_. Briefly, Whole cells and supernatant were frozen at −80°C to lyse cells, samples were defrosted on ice, then serially diluted in Minimum Essential Medium Eagle (Wisent, St-Bruno, QC) supplemented with 3% heat-inactivated FBS (Wisent, St-Bruno, QC) and 0.1% gentamicin. Supernatants were not analysed separately in this series of experiments. Diluted virus was applied to Vero cells in 3% heat-inactivated FBS (Wisent, St-Bruno, QC) and 0.1% gentamicin in 96-well plates. The virus is incubated with the cells for 5 days at 37°C/5% CO_2_. Syncytium formation was scored and TCID_50_ was calculated using the Karber method [90,91].

## Elisa

U937 cells were infected at an MOI of 0.01 with the indicated virus. At 48 hours post-infection, supernatant IFNα1 and IFNβ were measured by ELISA (PBL Interferon Source, Piscataway, NJ) as per the manufacturer’s instructions.

## Competing interests

The authors have no competing interest to declare.

## Authors’ contributions

KJS designed, completed, and analysed experiments, drafted and reviewed the manuscript; CT and LR designed and completed experiments; TZD and KHR drafted the model figure and revised the manuscript; WHM and BJW designed experiments and critically reviewed the manuscript. All authors read and approved the final manuscript.
